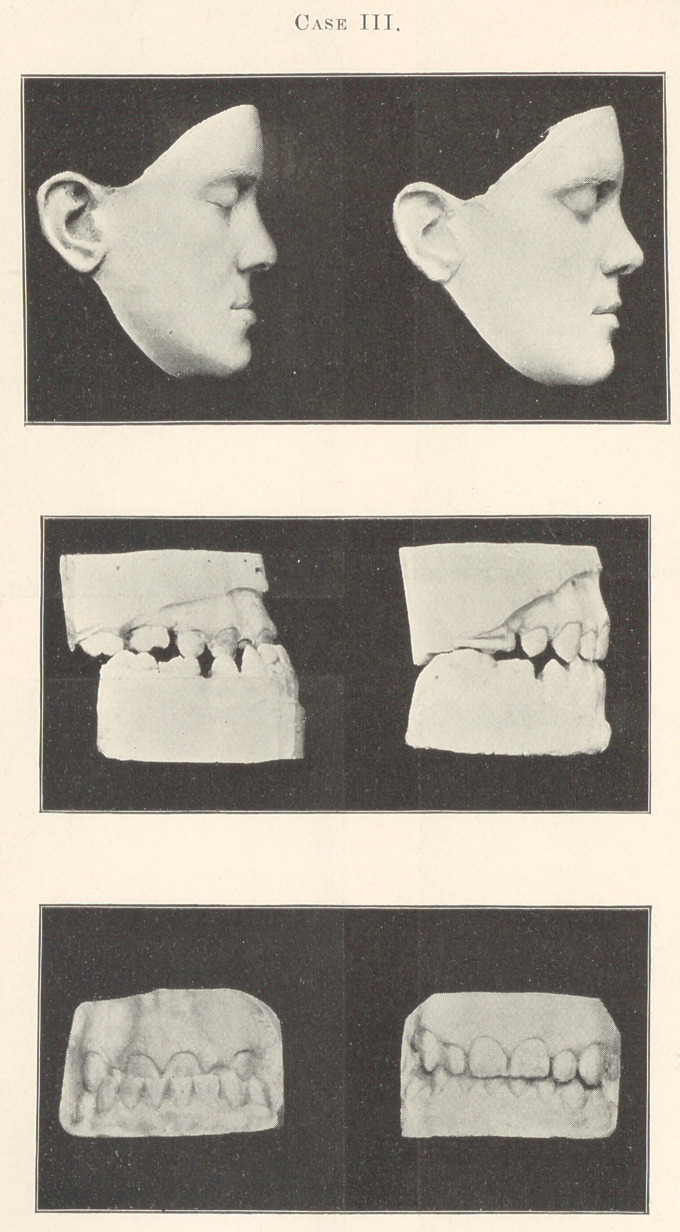# Dental and Facial Orthopædia

**Published:** 1895-08

**Authors:** Calvin S. Case

**Affiliations:** Chicago


THE
### International Dental Journal.

Vol. XVI. August, 1895. No. 8.

### Original Communications.[1]

### DENTAL AND FACIAL ORTHOPÆDIA.[2]

BY CALVIN S. CASE, D.D.S., M.D., CHICAGO.

In accepting the invitation of your Executive Committee to read a paper at this meeting, I have come with no expectation of presenting ideas that are entirely new, or that have not been presented by me and published in various places; but rather with the hope that I may more thoroughly convince you, by a careful examination of my models, of the importance and practicability of certain possibilities in applying force to the teeth, that will enable us, as dentists, to correct many imperfections and deformities of the face that have heretofore been considered impossible. I trust you will not consider this branch of facial orthopædia beyond the province of our profession, when you remember the wonders that have already been accomplished in this and every auxiliary department of dental science, and which have done much within the past few years to increase the scope of our professional attainments, beyond the mechanical practice of filling and inserting artificial teeth, to a position of the highest honor among the sciences.

Orthopædic surgery, as you are aware, is that branch of surgical

---

[1] The editor and publishers are not responsible for the views of authors of papers published in this department, nor for any claim to novelty, or otherwise, that may be mada by them. No papers will be received for this department that have appeared in any other journal published in the country.

[2] Read before the New York Odontological Society, March 19, 1895.

science which has for its object the correction of deformities. As it deals largely with those deformities of youth which are the result of an imperfect development of the osseous frame, the operations, as in orthodontia, mainly consist in the employment of mechanical apparatus, with the view of forcing nature to a restoration or production of a more natural form by a gradual and systematic application of force. Unlike orthodontia, however, one of the greatest difficulties which confronts the orthopædic surgeon, where pressure, tension, or torsion is indicated in particular deformities, is the impossibility of attaching the force appliances directly to the bones themselves, or without the intervention of soft, movable, and sensitive tissues. The limited power of the tissues to withstand continued pressure without the production of serious complications has given rise to a series of ingenious appliances and methods, of which manual and manipulative force play no unimportant part.

Besides the force of inheritance and diseases which produce deformities, it is a well-known fact that in certain vicissitudes of infantile and youthful life where particular parts of the body have been subjected to continued unnatural strain or pressure, the bones are often markedly deflected in their development and growth from assuming their natural form. And it is also as well known in orthopædic surgery that during the ages between five and ten, and often as late as fifteen or eighteen years, the bones yield to the slightest mechanical force, where it can be applied and continued in the direction of the desired movement. This is doubtless due to the cartilaginous condition of the bones before maturity, many of which, according to our best anatomists, are not completely ossified until after the age of eighteen or twenty. (See "Selection and Physical Examination of Recruits," by Charles B. Ewing, M.D., captain and assistant surgeon U.S.A. *Medical Record*, March, 1894.)

Notwithstanding the fact that the bones of the head are subject to the same laws of growth and development as are other bones of the body,—with the possible difference that they mature earlier,—little has been accomplished thus far in general orthopædic surgery for the correction of those facial deformities which are due to an imperfect shape or position of the facial bones; and still less for the perfection of æsthetic facial contours where the natural or inherited condition could hardly be called a deformity, though producing an expression of the features of the face far from attractive. In a conversation with a very prominent orthopædic surgeon in Chicago a few days ago, I asked what had been done in general orthopædia for the correction of deformities of the face. He said actually nothing had been done, except in very rare instances,—the practice being confined to accidental conditions almost entirely.

This is due, no doubt, principally to the difficulties heretofore presented for applying force to the bones of the face during their growth and development that would tend to give a more perfect facial form,—and it is partly due also to our habit of belief that the features of the face are stamped by inheritance or by the Creator, as they shall or must remain.

However, had some practical and sure method been discovered to accomplish this result, it would soon have eradicated all superstitious notions, considering the natural desire for æsthetic facial perfection so prominent in every one, and particularly with parents for their children.

It is a noteworthy fact that a very little change in the peripheral shape or position of certain bones of the face upon which the features are dependent for their character and form,—a change so trifling it could hardly be measured,—resulting in a slight filling out or depression of certain contours, will often beautify the appearance of a face that would otherwise be quite plain and unattractive. As a large proportion of facial imperfections and deformities are due to malposed teeth and jaws, to the dental profession is due the honor of almost everything that has been accomplished so far in facial orthopædia; and as the practice has been confined principally to the correction of irregularities of the teeth, it has given rise to that specialty of orthopædia known as orthodontia, which has been for years considered one of the proper branches of dentistry.

The term *orthodontia*, however, is not sufficient in itself, for its meaning is limited to irregularities of the teeth and their correction; whereas a movement of other parts quite as important in the reduction of certain facial deformities as the movement of teeth has long been recognized as within the possibilities of dental force appliances. And as this branch of the science is becoming more and more an important factor under the influence of modern methods, I would suggest as a more comprehensive and applicable title the term Dental Orthopædia.

The force which has from time to time been applied for the movement of malposed teeth has often changed the shape and surface contour of the alveolus in which the roots of the moving teeth were embedded,—and this latter movement in many instances has doubtless aided materially in the æsthetic facial effect of the operation. This is especially true where the dental arch superior or inferior has been expanded; also in the reduction of an anterior superior dental protrusion,—in the latter instance more particularly when occipital pressure has been used. In many instances the force of this movement being communicated to the more solid, though immature, structure of the maxillary and other bones, has doubtless exerted more or less influence in changing their general physical forms, with results that could not have been obtained by a movement of the teeth alone.

The change also which has been accomplished by permanently forcing the lower jaw to a more anterior or posterior position in its relations to the upper is quite remarkable, and important in facial orthopædia.

In this connection, I am pleased to call attention to a paper by Dr. Norman Kingsley, entitled "Adenoid Growths, Mouth-Breathing, and Thumb-Sucking in their Relations to Deformities of the Jaws and Irregular Teeth," read at the January, 1892, meeting of the First District Dental Society of this city, and published in several numbers of the *Dental Cosmos* of that year, in which the author has given us a number of beautiful illustrations of his undoubted skill in this branch of facial orthopædic science.

The limited area upon which force can be applied to a tooth compared to the portion covered by the gum and embedded in a bony socket has made it next to impossible, with all ordinary methods, to move the apex of the root in the direction of the applied force. Nor could this ever be accomplished with force applied in the ordinary way at one point upon the crown, however near the gum it be attached; for the fulcrum in that case would always be the opposing margin of the alveolar socket, with a tendency to move the point of the root in an opposite direction. But if the fulcrum be transferred from the margin of the alveolar socket to a point upon the tooth near the occluding portion of the crown, and the power applied at a point as far upon the root as the mechanical opportunities of the case will permit, the apparatus becomes a lever of the third kind, the power being directed to a movement of the entire root in the direction of the applied force.

This principle of force which, I believe, was first outlined by Dr. Farrar, of this city, for the lateral movement of the entire tooth, will be found of great advantage when applied to the anterior superior teeth for the reduction of those facial deformities that are due to an abnormal prominence or depression of that portion of the superior maxilla which supports the upper lip and lower portion of the nose. When force is directed in this way at right angles with the peripheral surface of the bone, and applied equally to a number of teeth standing side by side with roots embedded deeply in the alveolus, surrounded and more or less firmly connected with the cortical portion of true bone, there is usually little or no apparent absorption of the sockets as ordinarily occurs in the movement of a single tooth, but the immediate surrounding bony structure is carried bodily with the roots.

At the meeting of the World's Columbian Dental Congress, I presented a paper which will be found in the published proceedings of that Society, entitled "Some Principles governing the Development of Facial Contours in the Practice of Orthodontia." This paper was illustrated with plaster models of six cases in practice, covering the various facial imperfections and deformities produced by a malposition of the teeth and jaws, with a detailed description of different methods of applying the reciprocating principle of force in the reduction or development of facial contours. The purpose of the paper, as there stated, was to show how, with our present possibilities in the construction of dental regulating appliances, this principle of force could be applied to the forward or backward movement of the roots of the anterior teeth; and to illustrate also the importance of this possibility, when it is observed in this operation that the bones of youth do not remain stationary, to be ploughed through by the roots in a process of retrogressive metamorphosis, but that a considerable portion of the bone in which the teeth are embedded is carried with the roots in proportion as they are changed in position, thus enabling one to regulate many imperfections of the face by changing the shape and surface contour of the frame which supports and gives character to the features, over all that portion which can be effected by a movement of the bones contiguous to the roots of the teeth.

I have brought for your examination the models of six cases which have not been published elsewhere. In two of these I have finished treatment, one is nearly finished, and the balance are in different stages of completion. I have selected these with a view of showing the typical character of irregularity which often comes under your observation, that is accompanied almost invariably by a facial defect that is caused by the malposition of the roots of the anterior teeth and the surrounding bony structure, upon which important features of the face depend for their character. I have also brought the models of two cases that were exhibited at the Dental Congress, thinking you may not have had an opportunity there, in the rush of other things of importance, to personally or carefully examine them.

I wish to call your attention, right here, to the discussion which followed the reading of my paper at the Congress, in which a number of prominent dentists who, having seen a number of the patients whose models were there exhibited, kindly stated that the models did not do justice to the improvement I had accomplished. It is true that these models, which are made from exact plaster impressions of faces more or less deformed, do not fully express imperfections that would be markedly apparent by a personal observation of the face itself. The same is true of photographs which express but one, and usually the best, of many facial attitudes. And, on the other hand, while these imperfections are not usually so noticeable to friends and relatives, the slightest improvement towards beautifying the face by a removal of those unpleasant aspects, so apparent to strangers, is quickly noticed and even exaggerated by all who have had an opportunity to observe the various expressions of the face before and after treatment.

Before pointing out certain features of each particular case which I wish you to observe in your examination of the models, I will first explain in an informal talk the most recent method I have adopted in the construction and application of the dental contouring apparatus. In viewing this apparatus for the first time in the mouth or elsewhere, the first thought is in regard to its extent, apparent complication, and the difficulties in its construction and practical application; and the second is in regard to the pain and annoyance it must cause to the patient.

Of the latter, I wish to say, I have at present eleven patients, each wearing an apparatus of this character. All, except one, are attending school without the slightest interference or interruption of their school or other requirements, nor special inconvenience or irritation of the gums and mucous membrane. One of these patients, as Dr. George Cushing will tell you, is a boy of an exceedingly nervous temperament, having been affected for years with a mild type of chorea or St. Vitus's Dance, from which he has not fully recovered. When I commenced treatment this was one of the most pronounced cases of inlocked superior incisors I have seen with marked facial imperfection, characterized, as is common in these cases, with a decided depression of the middle features of the face, prominent lower lip, and apparent protrusion of the chin. (See Case III.) Having been reared with all the gentle care and luxury which wealth can give, and also because of his infirmity, it was eminently desirable to avoid any continued disturbance to his nervous system. He has now worn the apparatus over three months and will soon be ready for the staying bands, with a result that will be exceedingly satisfactory. During this time he has not lost over three or four days of school, which is usually not necessary, and with no apparent increase of his nervous disorder.

In the construction and application of the contouring apparatus, I experienced many discouraging difficulties in my early attempts, notwithstanding the fact that in my first case I succeeded in reducing the most pronounced deformity of the face I have yet treated. (See letter from the father of this patient, published in proceedings of the World's Columbian Dental Congress, pp. 734, 735.) Now, all my former difficulties are reduced to a minimum, and the whole operation to a system that as nearly approaches an exact science as any other operation in dentistry.

The first case I wish to call your attention to comes under that class of facial deformities I have described elsewhere as a protrusion of the upper lip, at that point where it merges into the nasal septum and orifices, due to a malposition of the roots alone of the incisor teeth. This position of the roots of the superior incisors, with entire obliteration of the incisive fossæ, is not uncommon, even when the occluding ends of the teeth are in proper position, and often with the production of quite a marked facial deformity.

Case I.—Miss R., aged twenty-two, commenced treatment December 17, 1893; staying bands October 3, 1894. The unusual time required for this operation was due to the age of the patient, and consequent impossibility of effecting a more rapid movement of the teeth. It will be observed by an examination of the models that the first superior bicuspids were removed and the space subsequently closed,—not so much by a backward movement of the crowns of the anterior teeth as by a forward movement of the molars, due to the great anchorage force they were obliged to sustain. It will also be observed by a careful examination that the cervical portion of the incisors have not materially changed their relative position, though the apices of their roots must have moved back at least three-eighths of an inch, judging from the present inclination of these teeth and the marked depression of the covering tissues, as compared to the first models.

The cuspids did not originally stand at the same inclination as the incisors, nevertheless it will be observed that the roots of these teeth, which always offer the greatest resistance, have moved appreciably. What to me is more to the point and of infinite importance, compared to the correction of the dental irregularity, is the remarkably beautifying effect this slight change has produced on the face of the patient, which, I must say, the models inadequately express. For further proof of this statement, I am pleased to be able to refer you to a man whom you all know, Dr. A. W. Harlan, who referred the case to me.

I have also here the models of another case to further illustrate this same type of deformity. (See Fig. 4.) I wish to say now in regard to this character of irregularity that experience has taught me that it is infinitely more difficult to move the roots and adjacent bone of the anterior superior teeth backward, especially that of the cuspid, than to move them forward, for reasons that are needless te explain. However, if the operation is performed as early in life as opportunities are afforded for attaching the appliances, and at a time when the superior maxillæ is more immature and cartilaginous, I believe that few difficulties will be experienced if the force is properly applied.

Case II.—Miss M., aged sixteen; commenced treatment December 26, 1893; staying bands October 15, 1894. In this case the upper jaw was too small for the teeth, which were greatly crowded, and with the cuspids, as will be seen, in their customary positions under these conditions. The dental arch was lacking in its anterior extension rather than in width, the incisors being quite posteriorly placed as regards the other teeth, producing a marked depression of the upper lip that was decidedly inharmonious, to say the least. In preparing it for the application of the contouring apparatus, the crowns of the incisor teeth were first forced forward with jackscrews, and the cuspids crowded down more nearly into alignment. At this stage in the operation models of the case were exhibited at the Illinois State Dental Society, to show the common facial result of the ordinary method of correcting this character of irregularity. The crowns of the incisor teeth were pushed forward at a considerable angle, and all the teeth were crowded, with contracted interproximate spaces. The incisive fossæ seemed deeper than ever, while the facial imperfection was unimproved. The model which you will examine of the teeth at this stage of correction is mounted with the contouring apparatus in place, similar to the one which was at this time attached to the teeth of the patient. Now, mark the change which occurred after wearing this apparatus four months, as shown in the final model of the teeth. Notice the upright position of the incisor teeth and the ample room that has been obtained for all the teeth, and, moreover, this change has produced, as in other instances, a decidedly favorable improvement to the face.

Case I.

Case II.

Case III.

Case III.—George T., aged fourteen, is the case from Dr. Cushing which I previously mentioned.[1]

By examining the models of the teeth it will be found that the superior laterals were badly rotated because of insufficient room between the cuspids that were inlocked by the buccal cusps of the inferior bicuspids. Before it was possible, therefore, to attach the contouring apparatus it was necessary to widen the space for the incisors and rotate the laterals into proper alignment. This proved to be a most difficult and trying part of the operation on account of the peculiar occlusion of the teeth, it requiring something over a month before they were ready for the contouring apparatus, which was finally placed on the teeth December 24, 1894, and removed March 14, that I might take impressions for the purpose of showing you what had been accomplished in this case after wearing the contouring apparatus about two and a half months.

I mention this with no intention of leading you to infer that this is the ordinary time these cases require, but rather as an unusual result.

Time, in my estimation, is a factor of the least importance in all orthopædic operations; although there are many who make a specialty of this branch of dentistry who seem to think they will not be considered skilful unless they can manage to make you wonder how it is possible they can accomplish this or that in so short a time. The facts are, that the cases which have every appearance of being the easiest of their class often prove to be the most obdurate, and *vice versa;* so that no one or a half dozen cases can be cited as an example of skill or of time that others will require. That which is of the most vital importance should lie in the possibility of accomplishing the result you aim at without injury to the teeth or to the health of the patient. It is also important that the apparatus which you devise should be mechanically and orthopædically correct, in order that it does its work as rapidly as the requirements of nature will permit, and with the least possible amount of pain or inconvenience to the patient.

The balance of the models, or Cases IV., V., and VI., which I exhibit here for the first time, are not completed, therefore I will reserve their publication,—having brought them, as I said before, to

---

[[1] At the Tri-State Dental Meeting, at Detroit, Dr. Cushing, in speaking of this case, said, "These models do not begin to express the great change which has been accomplished in this and other cases I have seen which Dr. Case has treated."—Ed.]

more fully exemplify the peculiar character of certain common types of deformities which are amenable to treatment with more than pleasing results. I have, however, taken modelling compound impressions of the anterior aspect of some of these cases with the jaws closed and the apparatus in place. From the models of these you will be able to judge of the present advancement of the different operations.

If I succeed in convincing you that this peculiar character of deformity demands at your hands a course of treatment that is capable of restoring to the face its natural beauty, and that in the future nothing short of this will satisfy you, I shall go home from this meeting with the proud and satisfied feeling that I have not lived in vain.

## Figures and Tables

**Case I. f1:**
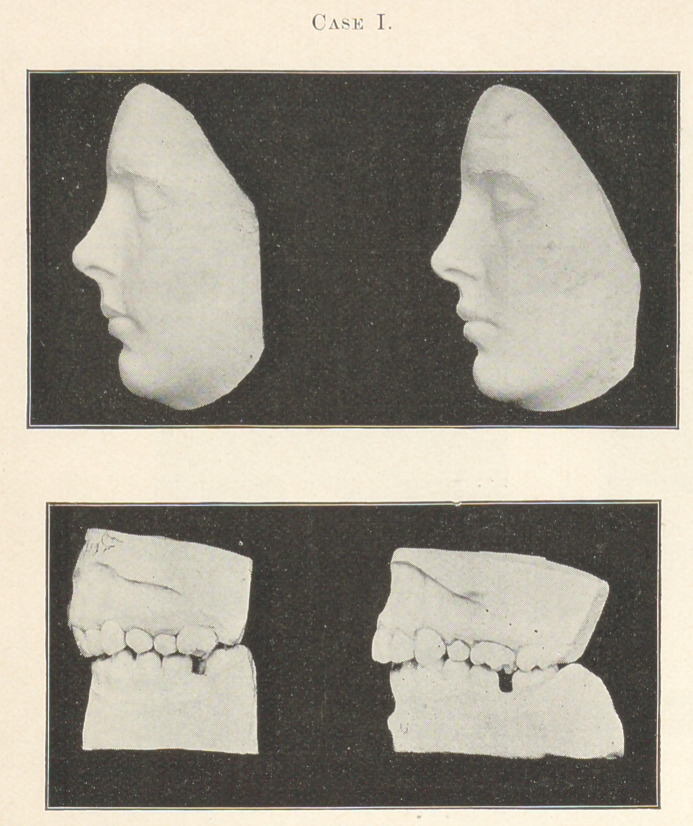


**Case II. f2:**
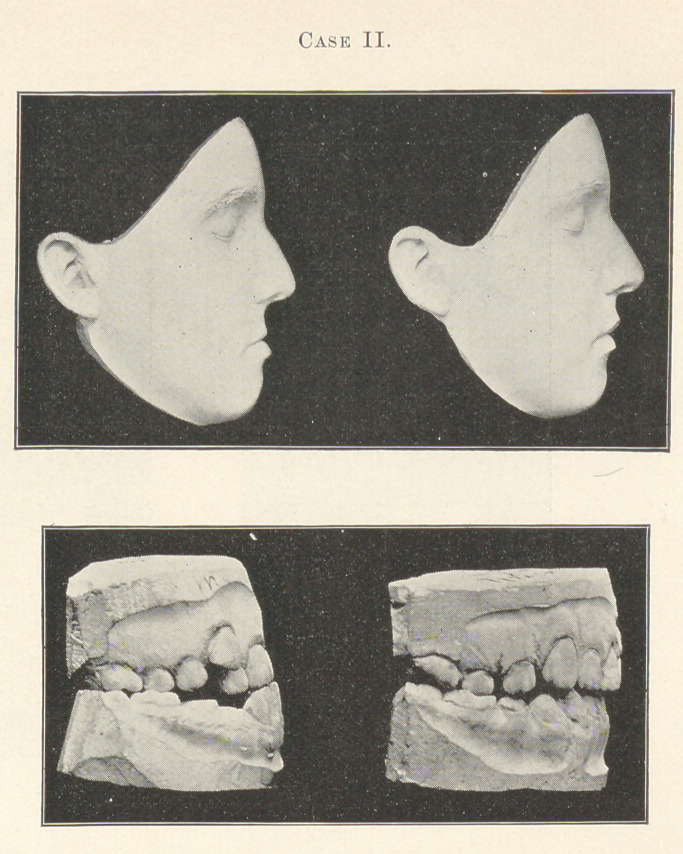


**Case III. f3:**